# Examining the Forearm Intersection through Palpation and Ultrasonography

**DOI:** 10.3390/diagnostics14010116

**Published:** 2024-01-04

**Authors:** Esperanza Naredo, Jorge Murillo-González, José Ramón Mérida Velasco, Otto Olivas Vergara, Robert A. Kalish, Cristina Gómez-Moreno, Eva García-Carpintero Blas, Gema Fuensalida-Novo, Juan J. Canoso

**Affiliations:** 1 Department of Rheumatology and Bone and Joint Research Unit, Hospital Universitario Fundación Jiménez Diaz, IIS Fundación Jiménez Díaz, Autónoma University, 28049 Madrid, Spain; enaredo@ser.es (E.N.); otto.olivas@quironsalud.es (O.O.V.); 2 Department of Anatomy and Embryology, Faculty of Medicine, Complutense University of Madrid, 28040 Madrid, Spain; mvlopera@med.ucm.es; 3 Division of Rheumatology, Tufts University School of Medicine, Boston, MA 02111, USA; rkalish@tuftsmedicalcenter.org (R.A.K.); jcanoso@gmail.com (J.J.C.); 4 Department of Nursing, Hospital Universitario Fundación Jiménez Díaz, 28049 Madrid, Spain; cgomezm@fjd.es (C.G.-M.); eva.garciab@quironsalud.es (E.G.-C.B.); gfuensalida@fjd.es (G.F.-N.); 5 Department of Medicine, Emeritus, ABC Medical Center, Mexico City 01120, Mexico

**Keywords:** forearm pain, tendon rub, forearm intersection, abductor pollicis longus, extensor pollicis brevis, extensor carpi radialis brevis, extensor carpi radialis longus

## Abstract

Background: Forearm intersection syndrome causes pain, swelling, and a rub at the dorsal distal forearm where the first extensor compartment muscles intersect with the second compartment tendons. Although primary care settings tend to treat mild cases, high-performance athletes may suffer from severe symptoms that require surgery. This proof-of-concept study aims to help detect the anatomical substrate of forearm intersection syndrome using palpation and ultrasonography when available. Methods: Five individuals were studied using independent palpation and ultrasonography to identify the first dorsal compartment muscles and the second dorsal compartment tendons. The distances between the dorsal (Lister’s) tubercle of the radius and the ulnar and radial edges of the first dorsal compartment muscles were measured to determine the location and extent of the muscle–tendon intersection. The palpatory and ultrasonographic measurements were compared using descriptive statistics and the paired *t*-test. Results: The mean distances from the dorsal tubercle of the radius to the ulnar and radial borders of the first dorsal compartment muscles were 4.0 cm (SE 0.42) and 7.7 cm (SE 0.56), respectively, based on palpation. By ultrasonography, the corresponding distances were 3.5 cm (SD 1.05, SE 0.47) and 7.0 cm (SD 1.41, SE 0.63). Both methods showed a similar overlap length. However, ultrasonography revealed a shorter distance between the dorsal tubercle of the radius and the ulnar border of the first compartment than palpation (*p* = 0.0249). Conclusions: Our findings indicate that a basic knowledge of anatomy should help health professionals diagnose forearm intersection syndrome through palpation and, if available, ultrasonography.

## 1. Introduction

A basic understanding of clinical anatomy is crucial to identifying tendinopathies in the distal upper extremity [1,2,3]. One common example of such tendinopathy is the trigger finger. It occurs when the bulky first annular pulley blocks the flexor tendons, which can also be thickened, below the transverse palmar creases [3]. On the other end of the spectrum, forearm intersection syndrome (FIS) is rare in general clinical practice but prevalent in sports medicine settings. This syndrome was first described by Velpeau in 1837 [4]. It occurs where the first extensor compartment muscles, extensor pollicis brevis (EPB), and abductor pollicis longus (APL) cross the deeper-seated second compartment tendons, extensor carpi radialis brevis (ECRB), and extensor carpi radialis longus (ECRL). FIS can be disabling for athletes involved in rowing, canoeing, skiing, weightlifting, and racket sports [5,6,7].

The ECRB and ECRL are two muscles located in the superficial extensor compartment of the arm, running along the lateral surface of the radius. They originate from the humerus and extend to the hand, passing under the extensor retinaculum in the second dorsal compartment, enclosed in a single synovial sheath. The ECRB muscle is inserted into the dorsum of the base of the third metacarpal, while the ECRL is attached to the base of the second metacarpal. The APL and EPB are two muscles located in the deep extensor compartment. They originate from the dorsal aspect of the ulna (EPB inconsistently), interosseous membrane, and radius, just distal to the anconeus and supinator muscles. The EPB originates distal to the APL and joins it, following the same trajectory and relationships. These muscles extend obliquely in a lateral and caudal direction and reach the hand under the extensor retinaculum in the first dorsal compartment in a single synovial sheath, which is wholly or partially septated in approximately 40% of cadaveric specimens [8]. These tendons are inserted on the thumb, with the APL on the lateral side of the first metacarpal base and the EPB on the dorsal side of the proximal phalanx. In the distal third of the forearm, they emerge between the extensor digitorum and the ECRB and cross over the tendons of the ECRB and ECRL without an interposed serous bursa [9,10]. FIS is characterized by pain, swelling, and often a tendon rub over the distal dorsal forearm [11]. Snapping is rarely felt [12]. The symptoms are mild or absent if the extremity is kept still but suddenly worsen by moving the thumb or deviating the wrist radially. Patients experiencing mild FIS, which justifies the current study, may seek consultation from general medicine, rheumatology, physiatry, or other professionals. Overuse tendinitis is often diagnosed in such cases, and treatment with a splint, a non-steroidal anti-inflammatory drug, and, occasionally, a corticosteroid infiltration is usually successful. However, an accurate diagnosis can be made through plain palpation or, if available, inexpensive ultrasonography at the point of care [13]. On the other hand, orthopedic surgeons or sports medicine specialists typically diagnose and treat more severe FIS in sports settings, and an MRI tailored to the clinical findings is required in these patients [5,7]. The literature on FIS in rheumatology and related journals is surprisingly meager. Suffice it to say that the authors reviewed thirty-two rheumatology journals listed in PubMed, finding only two Letters to the Editor [14,15]. In addition, among the twenty-seven physical medicine and rehabilitation journals, only four reports were found: one case report [9] and three case series [7,11,16].

Thus, understanding anatomy is crucial in identifying the site where FIS occurs. In this proof-of-concept study, palpation and ultrasonography were independently used to determine the crossing area. In addition, an anatomical dissection was performed to better understand the general area where FIS occurs. The authors intended to provide clinicians with enough anatomical information to allow an immediate clinical diagnosis, avoiding worries or the use of unnecessary expensive procedures such as MRIs, which are, however, key for treating severe cases. This view is inscribed in the overarching goal of improving clinicians’ diagnostic capacity based on clinical examination findings, with the possible addition of ultrasonography, in an age when clinical skills are decreasing and reliance on not-always-needed technology is increasing at a faster pace.

## 2. Materials and Methods

This study examined the dominant side of five Caucasian participants, comprising two females and three males aged between 40 and 62. None of them had any current or past pain, trauma, or surgery in their upper extremities, and they had not engaged in heavy sports or exercise on the study day. Four participants had a normal body mass index, while one was overweight. During the study, all participants were seated by an examining table with their elbows flexed 90 degrees and their palms resting on the table. The clinical and ultrasonography measurements were conducted independently of each other. Although two experienced anatomists (JMG and JRMV) provided general advice during the study, they were not made aware of the results of the measurements.

### 2.1. Palpatory Measurements

**Figure 1:**
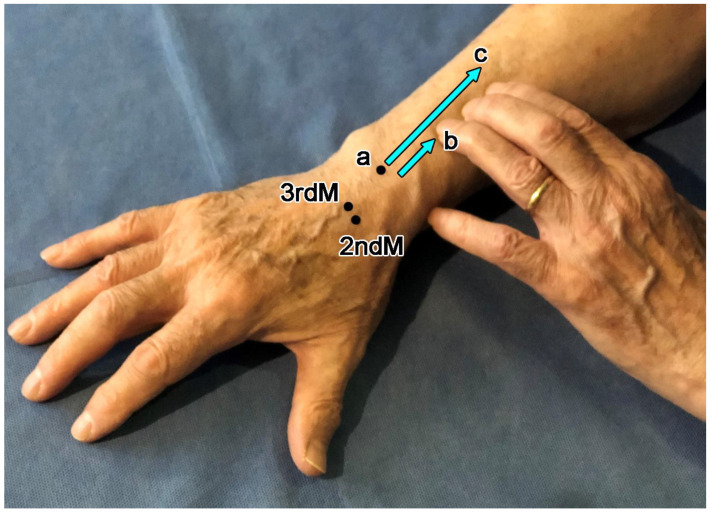
The landmarks used for palpation were the second and third metacarpal bases (2ndM, 3rdM), where extensor carpi radialis longus and extensor carpi radialis brevis are inserted. a, dorsal tubercle of the radius of radius; b, ulnar edge of extensor pollicis brevis; c, radial edge of abductor pollicis longus. Blue arrows indicate the distances between the dorsal tubercle of the radius and the ulnar and radial borders of the 1st compartment muscles.

During the clinical examination, a rheumatologist with more than 45 years of experience in musculoskeletal anatomy (JJC) palpated the dorsal surface of the distal end of the radius. He located the dorsal tubercle of the radius, which is adjacent to the radiocarpal joint as can be determined by flexion and extension movements of the wrist, and marked it with a soluble marker. The clinician then followed the tendons of ECRL and ECRB from their insertion at the base of the second and third metacarpals, respectively, proximally along the radius to the crossing ulnar edge of EPB and, more cephalad, the radial edge of APL (Figure 1). To identify the full extent of the crossing, the examiner placed his annular, middle, and index fingers along the radius, between the ulnar (distal) and radial (proximal) edges of the muscle layer, and asked the subject to repeatedly make minor radial deviations of the wrist to tense the superficially placed EPB and APL muscles and repeatedly make slight dorsiflexions of the wrist to tense the deeper-seated ECRB and ECRL tendons (Figure 1). Two measurements were taken along the dorsal radius: one from the distal edge of the dorsal tubercle of the radius (a) to the ulnar oblique border of EPB (b), and the other from the dorsal tubercle of the radius (a) to the radial oblique edge of APL (c). The difference between these two measurements was the length of the muscle–tendon overlap.

### 2.2. Ultrasonographic Measurements

Two experts (EN, OO-V), with 27 and 5 years of experience in musculoskeletal ultrasound, respectively, scanned the ECRB and ECRL tendons in the short (transverse) axis from the dorsal tubercle of the radius distally to their musculotendinous junction proximally. Along this path, the overlying muscles EPB and APL were encountered. A real-time scanner (LOGIQ E10, GE Medical Systems Ultrasound and Primary Care Diagnostics, LLC, Wauwatosa, WI, USA) was used with a multifrequency linear transducer (ML 6–15 MHZ). B-mode settings were standardized for the study: B-mode frequency 15 MHz, B-mode gain 60 dB, and dynamic range 66 dB. As in the palpatory examination, the distance from the distal edge of the dorsal tubercle of the radius to the ulnar border of EPB and the distance from the dorsal tubercle of the radius to the radial edge of APL were measured, marking on the skin the location of these structures when they were visualized by ultrasound. In addition, videos were obtained of the distal–proximal ultrasound sweep and the crossing between the tendons of the second dorsal compartment and the muscles of the first dorsal compartment during repeated dorsiflexion and radial deviations of the wrist.

### 2.3. Institutional Approval

The clinical and ultrasonographic study was approved by the Ethics Committee of the Jiménez Díaz Foundation, project 28-06-2022, PIC118-22_FJD, and signed informed consent was obtained from all subjects.

### 2.4. Dissection Study

The dissection study was performed in accordance with the Declaration of Helsinki. The corpse belonged to the Center of Donation of Corpses, Complutense University of Madrid. All local and international ethical guidelines and laws regarding using human cadaveric donors in anatomical research were followed. Before death, all individuals gave written, informed consent to use their donations for scientific purposes. During our study, we examined six preserved upper limbs, three left and three right, from six different bodies (three male and three female, aged between 69 and 98 years at the time of death). In one of the dissections, we identified the regional anatomy and landmarks used in our measurements. In the other five dissections, we focused on the area where the muscles of the first and second extensor compartments cross, in an attempt to locate a serous bursa.

## 3. Results

The measurements from palpation are shown in Table 1. By palpation, the mean distance from the dorsal tubercle of the radius (a) to the ulnar edge of the EPB (b) had a median of 4.0 cm (SD 0.94, SE 0.42). The corresponding distance to the radial edge of the APL (c) was 7.5 cm (SD 1.25, SE 0.56). By ultrasonography, the corresponding distances were 3.5 cm (SD 1.05, SE 0.47) and 7.0 cm (SD 1.41, SE 0.63). Ultrasonography revealed a shorter distance between the dorsal tubercle of the radius and the ulnar border of the first compartment than palpation (*p* = 0.0249).

The overlap between the muscles of the first compartment and the tendons of the second compartment had a median of 3.5 cm (SD 1.04, SE 0.46) by palpation and 3.7 cm (SD 1.48, SE 0.66) by ultrasound. Overall, the general area formed a parallelogram with two parallel sides running along the radius, the ECRB and ECRL tendons, and two oblique parallel sides, the EPB and APL, running from ulnar to radial and proximal to distal across the radius, superficial to the tendons. Therefore, when moving proximally from the dorsal tubercle of the radius, the first muscle the examiner comes across is the ulnar edge of the EPB, and the most proximal corresponds to the radial edge of the APL.

**Figure 2:**
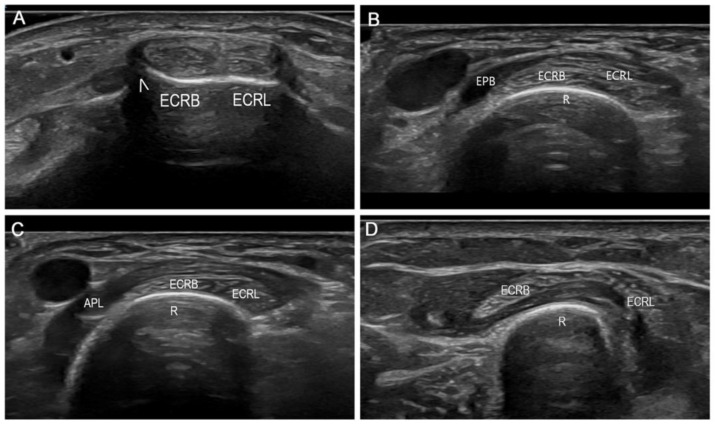
Ultrasound scanning of the second dorsal compartment tendons and the first dorsal compartment muscles, short-axis view. (**A**) Extensor carpi radialis brevis (ECRB) and extensor carpi radialis longus (ECRL) at distal dorsal tubercle of the radius level (arrowhead). (**B**) Crossing between the second dorsal compartment tendons (ECRB, ECRL) and the extensor pollicis brevis (EPB) muscle. R, radius. (**C**) Crossing between the second dorsal compartment tendons (ECRB, ECRL) and the abductor pollicis longus (APL) muscle. R, radius. (**D**) Musculotendinous junction of the ECRB and ECRL. R, radius.

Figure 2 illustrates the ultrasound scanning of the ECRB and ECRL tendons from the dorsal tubercle of the radius to their tendomuscular junction, revealing their crossing with the overlying first dorsal compartment muscles. While palpation allowed the identification of only one subject’s two muscles in the first compartment and two tendons in the second compartment, ultrasound enabled differentiation in all subjects. Videos S1–S4 demonstrate the dynamic ultrasound scans and changes at the intersection site for better clarity.

**Figure 3:**
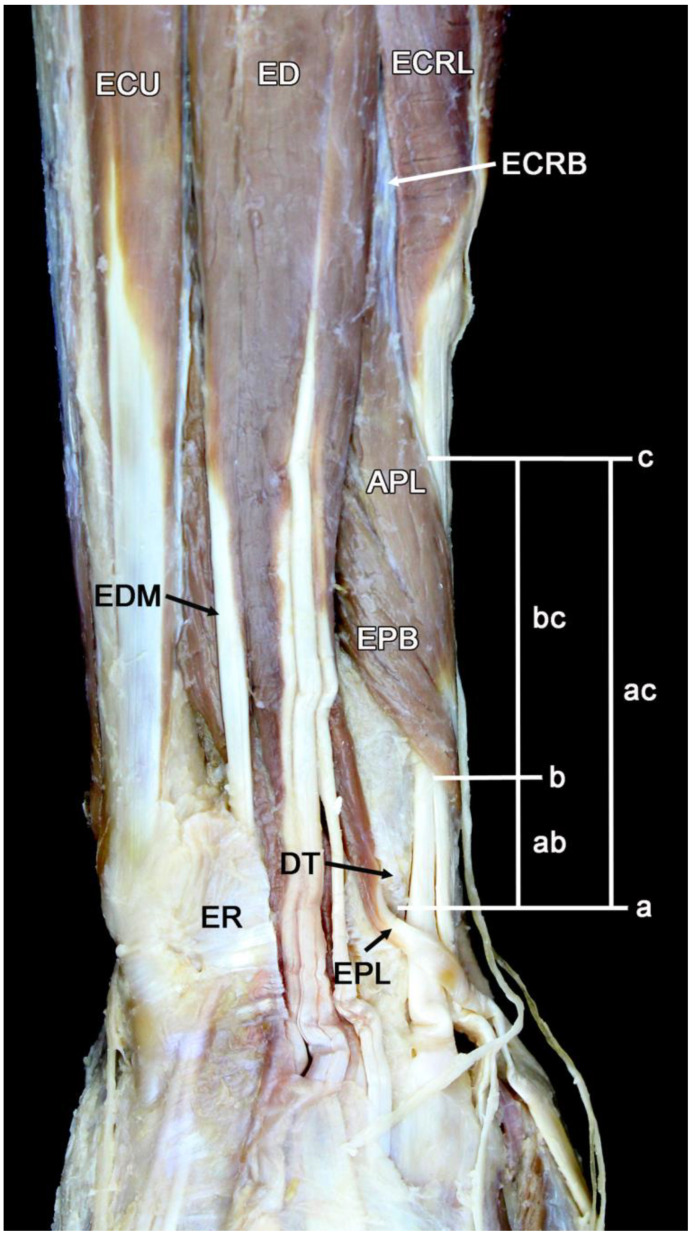
Dorsal dissection of the distal forearm and wrist. a, horizontal line indicating dorsal tubercle (DT) of the radius; b, horizontal line indicating the ulnar edge of the extensor pollicis brevis (EPB); c, horizontal line indicating the radial border of the abductor pollicis longus (APL); ab, distance from DT to the ulnar edge of EPB; ac, distance from DT to the radial edge of APL; bc, the overlap between the muscles of the first compartment (APL, EPB) and the tendons of the second compartment (ECRL, extensor carpi radialis longus; ECRB, extensor carpi radialis brevis). ECU, extensor carpi ulnaris; ED, extensor digitorum; EDM, extensor digiti minimi; EPL, extensor pollicis longus; ER, extensor retinaculum.

Figure 3 is a cadaveric dissection showing the dorsal tubercle’s location and the intersection of the APL and EPB over the ECRB and ECRL tendons. The oblique course of the muscle bellies over the tendons is from ulnar to radial and from proximal to distal. This dissected dorsal distal forearm and wrist show the landmarks and tracings of the palpatory and ultrasonographic measurements.

During the examination of the area where the muscles of the first and second extensor compartments cross, we observed the tendon sheaths of the ECRB and ECRL muscles. However, we were unable to find any evidence of a serous bursa.

## 4. Discussion

FIS is characterized by pain, swelling, and a tendon rub felt over the dorsal radial distal forearm area. The two bony landmarks that assist in diagnosing FIS are readily palpable. One is the flat dorsal distal head of the radius, and the other is the dorsal tubercle of the radius, located distally on the flat surface of the radius adjacent to the radiocarpal joint. Identifying the dorsal tubercle of the radius was emphasized in a Delphi study of anatomical structures useful to rheumatologists [17]. Pantukosit’s fascinating report [8] observed that all but one of 30 FIS cases occurred on the dominant side, and most of the patients were rice farmers who performed repetitive, forceful wrist movements. All the patients reported pain, while 22 experienced swelling and 12 had a tendon rub. Most mild cases of FIS occur after occasional or repetitive excessive exercise with the upper extremity. Unless the entity is known, cases go undiagnosed or misdiagnosed as unspecified tenosynovitis.

For the reasons mentioned above, ultrasonography is a non-invasive imaging technique increasingly used to optimize the diagnosis and treatment of musculoskeletal diseases. This imaging modality, although operator-dependent [18], is an accessible tool that can be performed in the same consultation room, allowing immediate correlation with the patient. It is safe, relatively inexpensive compared to other imaging techniques, quick in expert hands, and very well accepted by the patient. One of its unique advantages is that it allows the dynamic–functional study of musculoskeletal structures. Ultrasonography has been extensively validated for assessing superficial tendons and muscles [19,20,21].

The actual forces involved in the pathogenesis of intersection syndrome are just beginning to be understood. In a recent study, the investigators placed a force sensor between the first compartment muscles, the EPB and APL, and the radius in cadaveric specimens and moved the wrists in palmar flexion, extension, pronation, supination, and radial and ulnar deviation. The highest pressures on the distal radius were recorded in pronation and ulnar deviation without flexion/dorsiflexion [22].

Distal intersection syndrome, entirely different from FIS, occurs in the wrist rather than the forearm, just distal to and radial to the dorsal tubercle of the radius. At this tubercle, the extensor pollicis longus tendon turns radially towards the thumb and covers the ECRB and ECRL tendons; this crossing may lead to synovitis if overused, such as when playing drums [23]. Findings include pain and swelling in the dorsal–radial wrist, which can be confused with radial wrist synovitis. The relative frequency of FIS and distal intersection syndrome may be surmised from a detailed study of 1131 patients with hand and wrist US examinations focusing on those with intersection syndrome. Of 15 male patients, 2 had wrist intersection syndrome and 13 had FIS; of 6 female patients, 1 had wrist intersection syndrome and 5 had FIS. Intersection syndrome was bilateral in one patient, and the condition predominated, four to one, on the dominant side. Ultrasonography is ideally suited to document a diagnosis of FIS and distal intersection syndrome [13].

The differential diagnosis of FIS includes several conditions (Table 2). Based on local findings, infection and scleroderma may be considered. The absence of any abrasion, puncture, or other entry point makes soft-tissue infection unlikely. Likewise, scleroderma is highly unlikely because only one side is affected, there is no Raynaud’s phenomenon, and there are no nail fold vascular changes. FIS must be distinguished from other conditions, including de Quervain’s tenosynovitis [24], superficial sensory radial nerve neuropathy due to nerve damage or external pressure (Wartenberg syndrome) [25,26,27], osteoarthritis of the first carpometacarpal joint, and intersection syndrome at the wrist [3,13,14,25,27,28,29]. Table 2 summarizes the overlapping features and differences between these conditions.

The extensive swelling in FIS is explained by the oblique course of the APL and EPB over the wrist extensors. FIS can also cause subcutaneous edema beyond the anatomical intersection limits. The crossing area can extend from 2.5 to 9 cm from the dorsal tubercle of the radius, which means that only the proximal portion of this area is usually included in a routine wrist MRI protocol. A study [30] that used detailed MRI, tenography, and anatomy found that even when the MRI wrist protocol was extended to 7–7.5 cm, the proximal border of the intersection was missed. Another MRI study [31], which analyzed six patients, found that all patients had tendinitis, peritendinous edema, or fluid, and, with a decreasing frequency, muscular edema, subcutaneous edema that could extend proximally and distally to cover the extensor retinaculum, and juxtacortical edema. According to the authors of this study, clinical findings, rather than pre-set MRI guidelines, should dictate the extent of the MRI in cases where this expensive study is necessary. While classic anatomists [32,33] described a large bursa where crepitations could arise between the muscles of the first compartment and the tendons of the second compartment, recent anatomy textbooks do not mention such a bursa or even a synovial tendon sheath [9,10]. However, detailed US studies in FIS show a synovial sheath, probably adventitious and caused by the repetitive stress resulting in FIS. Ultrasound and MRI studies on asymptomatic elite rowers revealed edema around the tendons of the second compartment but not around the hypertrophic muscles of the first compartment. Additionally, no enlarged bursa was found in patients who required surgery [7]. These patients showed severe hypertrophy of the first compartment muscles, adventitious synovitis around the tendons, and synovial perforation [5,7].

The medical treatment of FIS is multifactorial. Improved tasks and tool design aim for a secure grip without wrist deviations. Non-operative treatment options include modified sports and work activities, ice packs, taping, resting splints with 15-degree extension, oral non-steroidal anti-inflammatory agents, and, on occasion, a local injection of a depo-corticosteroid in the second dorsal compartment [11,16].

The weaknesses of this study include the small number of patients, which could not be logistically solved, and a 1 cm discrepancy between the detection of the ulnar edge of the first compartment muscles in favor of ultrasonography. It is possible that the echoes of the ultrasound waves are more readily perceived than the haptic properties of the muscle edge. However, there were no differences in the extent of the overlap or the detection of the radial border.

In summary, the authors suggest a simple physical examination technique, with the desirable addition of ultrasound, that would help diagnose FIS, even in cases that may have gone unrecognized.

## Figures and Tables

**Table 1:** The forearm dorsal intersection by palpation and ultrasonography.

	Subject Number				
	1	2	3	4	5
Distances	Palpation (cm)				
ab *	4.0	2.5	4.0	5.0	4.5
ac **	9.5	6.0	7.5	8.0	7.5
bc ***	5.5	3.5	3.5	3.0	3.0
	Ultrasonography (cm)				
ab *	3.0	2.5	3.0	4.0	4.0
ac **	9.0	6.0	6.0	6.0	8.0
bc ***	6.0	3.5	3.0	2.0	4.0

ab, distance from dorsal tubercle of the radius to the ulnar edge of EPB; ac, distance from dorsal tubercle of the radius to the radial edge of APL; bc, overlap between the muscles of the first compartment (EPB, APL) and the tendons of the second compartment (ECRL, ECRB); ab *, paired *t*-test *p* = 0.0249; ac **, paired *t*-test *p* = 0.2056; bc ***, paired *t*-test *p* = 1.0.

**Table 2:** Differential diagnosis of forearm intersection syndrome (FIS).

Forearm Intersection Syndrome	de Quervain Disease	Iatrogenic Damage of the Superficial Branch of the Radial Nerve	Compression Neuropathy of the Superficial Branch of the Radial Nerve (Wartenberg)	Trapezium- Metacarpal Osteoarthritis	Wrist Intersection Syndrome
Mechanism					
Overexercise (i.e., rowing); crossing of APL and EPB on ECRB and ECRL	Excessive lifting with thumb up (i.e., newborn baby)	Perineural adhesions or neuroma (i.e., following the release of de Quervain disease)	Pressure from a bracelet, wristwatch, or handcuff	Cartilage degradation and bone overgrowth	Overexercise (i.e., drum player); crossing of EPL on E and ECRL
Swelling					
Variable, dorsal forearm	At the radial styloid and radial wrist	No	No	Bony distortion	At the radial dorsal wrist
“Electric” pain					
No	No	Yes, radial–dorsal hand, thumb, index, middle, and annular fingers	Yes, radial–dorsal hand, thumb, index, middle, and annular fingers	No	No
Paresthesia					
No	No	Yes, as above	Yes, as above	No	No
Decreased sensation					
No	No	Yes, as above	Occasional	No	No
Neuropathic pain					
No	No	Frequent, as above	No	No	No
Tinel sign					
Negative	Negative	Positive	Positive	Negative	Negative
Thumb Finkelstein test *					
Positive (pulls APL, EPB)	Positive (pulls EPB)	Positive (pulls from a damaged nerve)	Positive (pulls from a compressed nerve)	Frequent (moves a distorted joint)	Positive (pulls from a pressing EPL)
Index Finkelstein test **					
Negative	Negative	Negative	Negative	Negative	Negative

APL = abductor pollicis longus, ECRB = extensor carpi radialis brevis, ECRL = extensor carpi radialis longus, EPB = extensor pollicis brevis, EPL = extensor pollicis longus. * Hand perpendicular to the edge of the table. The patient’s thumb is grasped, and the wrist is deviated ulnarward. ** Hand perpendicular to the edge of the table. The patient’s thumb is grasped, and the wrist is deviated ulnarward.

## Data Availability

The data presented in this study are available on request due to privacy restrictions from the corresponding author.

## Supplementary Materials

The following supporting information can be downloaded at: https://www.mdpi.com/article/10.3390/diagnostics14010116/s1, Video S1: Ultrasound scanning of the second dorsal compartment from the dorsal tubercle of the radius to the musculotendinous junction of the extensor carpi radialis brevis and extensor carpi radialis longus, passing through the intersection area with the first dorsal compartment muscles. Video S2: Second dorsal compartment tendons and overlaying abductor pollicis longus muscle during repeated radial deviations of the wrist. Video S3: Second dorsal compartment tendons and overlaying extensor pollicis brevis muscle during repeated wrist dorsiflexion. Video S4: Second dorsal compartment tendons and overlaying abductor pollicis longus muscle during alternating radial deviation and dorsiflexion of the wrist.
